# Hospitalizations among family members increase the risk of MRSA infection in a household

**DOI:** 10.1017/ice.2024.106

**Published:** 2024-07

**Authors:** Aaron C. Miller, Alan T. Arakkal, Daniel K. Sewell, Alberto M. Segre, Bijaya Adhikari, Philip M. Polgreen, For The CDC MInD-Healthcare Group

**Affiliations:** 1 Department of Internal Medicine, University of Iowa, Iowa City, IA, USA; 2 Department of Biostatistics, University of Iowa, Iowa City, IA, USA; 3 Department of Computer Science, University of Iowa, Iowa City, IA, USA

## Abstract

**Objective:**

Estimate the risk for household transmission of Methicillin-Resistant Staphylococcus aureus (MRSA) following exposure to infected family members or family members recently discharged from a hospital.

**Design:**

Analysis of monthly MRSA incidence from longitudinal insurance claims using the Merative MarketScan Commercial and Medicare (2001–2021) databases.

**Setting:**

Visits to inpatient, emergency department, and outpatient settings.

**Patients:**

Households with ≥2 family members enrolled in the same insurance plan for the entire month.

**Methods:**

We estimated a monthly incidence model, where enrollees were binned into monthly enrollment strata defined by demographic, patient, and exposure characteristics. Monthly incidence within each stratum was computed, and a regression analysis was used to estimate the incidence rate ratio (IRR) associated with household exposures of interest while accounting for potential confounding factors.

**Results:**

A total of 157,944,708 enrollees were included and 424,512 cases of MRSA were identified. Across all included enrollees, exposure to a family member with MRSA in the prior 30 days was associated with significantly increased risk of infection (IRR: 71.03 [95% CI, 67.73–74.50]). After removing enrollees who were hospitalized or exposed to a family member with MRSA, exposure to a family member who was recently discharged from the hospital was associated with increased risk of infection (IRR: 1.44 [95% CI, 1.39–1.49]) and the risk of infection increased with the duration of the family member’s hospital stay (*P* value < .001).

**Conclusions:**

Exposure to a recently hospitalized and discharged family member increased the risk of MRSA infection in a household even when the hospitalized family member was not diagnosed with MRSA.

## Background

*Staphylococcus aureus* is an important bacterial pathogen worldwide^1^ and a common cause of skin-and-soft-tissue infections.^2^ *S. aureus* also causes blood stream infections,^3^ osteomyelitis,^4^ and device-related infections.^5^ While *S. aureus* has been recognized as an important pathogen for over a century,^6^ several decades ago methicillin-resistant *S. aureus* (MRSA) emerged as an important pathogen.^7^ Initially, MRSA infections were associated with healthcare settings,^8–10^ and MRSA was rarely recovered from individuals without exposure to healthcare.^11^ In the 1990s community-associated MRSA infections emerged.^7^ More recently, community-associated strains of MRSA are endemic in healthcare settings.^11^

Community-associated MRSA strains frequently cause infections in otherwise healthy individuals.^11^ These infections are predominantly associated with skin-and-soft-tissue infections,^12^ but can also cause invasive infections.^12,13^ Community-associated-MRSA strains are frequently spread from person to person and are often spread via asymptomatically colonized carriers.^10^ Outbreaks or clusters of MRSA infections typically occur in crowded environments including military barracks, prisons, daycare centers, and especially within household settings.^14^ Household settings appear to be a primary reservoir for MRSA infections in the community,^12^ and transmission models suggest that households are a major site for MRSA transmission.^15^

Risk factors for household spread of MRSA include contact with an infected household member,^16^ participating in the care of an infected household member,^17,18^ presence of younger children in the household,^12^ households with more people,^19^ and the presence of domesticated animals.^20^ Strains of *S. aureus* including MRSA are frequently transmitted through skin-to-skin contact with colonized or infected individuals.^21–23^ Frequently, MRSA transmission is sustained because both colonized and infected household members can pass strains back and forth in a “ping-pong”-like fashion.^24^ The household environment can also be an important risk factor for MRSA colonization and infection.^19^ MRSA can be recovered from a wide range of fomites, and the organism can survive on some household surfaces for weeks and even months.^25^

While the household environment can help sustain the spread of MRSA, it first needs to be introduced into a household. As community-associated strains now frequently spread within healthcare facilities,^11^ recently discharged family members diagnosed with MRSA may be an initial source of infection. In addition, family members without an MRSA diagnosis who are asymptomatically colonized at discharge may be the causative factor for household spread. Also, because the risk of MRSA colonization is known to increase with length of stay,^26^ MRSA risk among non-hospitalized household members should increase with the recently discharged family member’s length of stay.

## Methods

### Study population

We used data from Merative Marketscan Commercial Claims and Encounters and Medicare Supplemental databases from 2001 through 2021. These databases represent over 240 million commercially insured enrollees in the United States, and contain claims from inpatient, outpatient, emergency department visits and outpatient prescription medications. Enrollment plan identifiers allow linkage of claims from multiple family members (ie, spouses, children, or dependents) enrolled in the same insurance plan. This study has been deemed non-human subjects research by the University of Iowa Institutional Review Board.

The study population was restricted to households where two or more family members could be identified on the same insurance plan and could be assumed to occupy the same household. Our analysis was based on monthly incidence, so we restricted our study population to enrollees continuously enrolled for an entire month. Cases of MRSA were identified in both outpatient and inpatient settings using the ICD-9-CM and ICD-10-CM codes shown in Supplementary Table 1. We focused on MRSA cases where the patient had no diagnosis of MRSA within the previous 30 days, to exclude cases of recurrent infections and/or subsequent care for the same infection.

### Statistical approach

#### Exposure to family member(s) with MRSA

The first objective was to evaluate the risk of developing MRSA associated with exposure to a family member with MRSA. We compared the monthly incidence of MRSA between individuals in households where another family member was recently diagnosed with MRSA to households with no recent family exposure to MRSA. Enrollees were defined as recently exposed to a family member with MRSA if another family member had an MRSA diagnosis (in an inpatient or outpatient setting) in the prior 30 days. For patients without MRSA, we used the start of a given month to identify family exposure within 30 days. For patients with MRSA, we considered exposure in the 30-day period prior to their initial MRSA diagnosis.

Enrollees were stratified into monthly enrollment strata based on the year and month, as well as the following demographic and patient characteristics: age (binned into 0–17, 18–40, 41–65, and >65 years), sex, comorbidities (total number of Elixhauser comorbidities in the prior 60 days), antimicrobial drug use within prior 60 days, presence of an infant <2 years of age in the household, hospitalization within the prior 30 days, exposure to a family member(s) with MRSA, and household size. A log-linear regression model was utilized to estimate the incidence rate ratios (IRRs) for the various patient strata while accounting for potential confounding effects. The following model was used to estimate the IRR associated with MRSA:

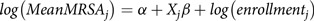




where for stratum *j*, 
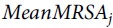
 is the expected count of the number of cases of MRSA in *j*, 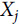 are the set of indicators that are used to define *j* (eg, prior exposure to a family member with MRSA, age bin, sex, comorbidities, etc), and 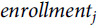 is an offset term used to control for the enrollment size of stratum *j*. A quasi-Poisson model was used to account for overdispersion. Note: This approach has been used to estimate the risk for secondary *Clostridioides difficile* infections among family members in household settings.^27,28^

#### Exposure to hospitalized family member(s)

For the second study objective, we evaluated the risk for asymptomatic household transmission associated with exposure to a family member who recently returned home from the hospital. We analyzed if exposure to a recently hospitalized and discharged family member who was not diagnosed with MRSA increased the risk of MRSA among household members following the discharge. To isolate the potential effect of asymptomatic household transmission attributable to hospitalization in a family member, we implemented two additional exclusion criteria to our study population to remove potential symptomatic exposures that may confound our results. First, we restricted our analysis to only those individuals that did not have a family member previously diagnosed with MRSA in the prior 30 days. Second, we further restricted our analysis to those enrollees who were not hospitalized themselves within the previous 30 days. We compared the monthly incidence of MRSA between individuals in households where another family member had been recently hospitalized within the previous 30 days to those without recently hospitalized family members. For this analysis, we included a single indicator in the model above if another family member was hospitalized in the prior 30 days. We used the log-linear regression model described above to estimate IRRs for the various patient strata.

We also analyzed whether the duration of time a family member spent hospitalized increased the risk of secondary transmission, under the hypothesis that longer hospital stays would be associated with greater risk of colonization. For this analysis, we computed the total amount of time that recently hospitalized family members spent in the hospital within the prior 30 days. We sum the lengths of stay across recently discharged family members’ inpatient stays where the discharge of the prior stay overlapped with the previous 30-day exposure window. For example, an individual with 2 family members hospitalized in the prior 30 days with a length of stay of 2 and 3 days, respectively, would have 5 total days of within-family hospitalization. Finally, we categorized total days of within-family hospitalization into bins (0, 1–3, 4–10, 11–20, 21–30, and >30 days), with the 0 days (ie, no hospitalization or a hospitalization of 0 days) of prior exposure as the reference bin. We then replaced the indicator for hospitalized family member in the log-linear model with the hospital stay duration categories.

##### Sensitivity analyses

We repeated both of our analyses (ie, exposure to a family member with MRSA and exposure to a recently discharged family member) using a 60-day window.

## Results

Baseline enrollment characteristics of the study population are presented in Table 1. From 2001 through 2021, a total of 157,944,708 enrollees were identified with two or more family members enrolled in the same insurance plan for an entire month. The majority of households contained four or more individuals in the same insurance plan (53.5%). A total of 424,512 MRSA cases were identified across 343,524 enrollees, of which the majority occurred among males (53.0%) and among enrollees aged 41 or older (57.2%). Of the MRSA cases we identified, 4,724 (1.1%) represented a possible transmission of MRSA that occurred after an MRSA diagnosis of a separate family member, while 8,064 (1.9%) represented possible transmission of MRSA that occurred after a hospitalization of a separate family member. Supplementary Figure 1 depicts trends in these different MRSA cases across time.

**Table 1.** Baseline enrollment characteristics of families with multiple household members

Characteristic	No. (%)			
	All enrollees	Episodes of MRSA diagnosis (events occurring ≥ 30 days prior to another episode)	Possible transmission following family member(s) MRSA diagnosis	Possible transmission following family member(s) hospitalization
**No. of cases**	–	424,512	4,724	8,064
**No. of enrollees**	157,944,708 (100)	343,524 (100)	4,568 (100)	7,891 (100)
**Age group at enrollment or MRSA diagnosis (years)**				
0-17	53,186,245 (33.7)	78,039 (18.4)	1,532 (32.4)	1,939 (24.0)
18-40	52,420,074 (33.2)	103,797 (24.5)	1,308 (27.7)	2,204 (27.3)
41-65	48,547,376 (30.7)	185,343 (43.7)	1,623 (34.4)	2,491 (30.9)
>65	3,791,013 (2.4)	57,333 (13.5)	261 (5.5)	1,430 (17.7)
**Sex**				
Male	78,264,337 (49.6)	224,856 (53.0)	2,383 (50.4)	3,999 (49.6)
Female	79,680,371 (50.4)	199,656 (47.0)	2,341 (49.6)	4,065 (50.4)
**Family size**				
2	40,247,824 (25.5)	187,234 (44.1)	1,089 (23.1)	3,093 (38.4)
3	33,285,360 (21.1)	84,628 (19.9)	899 (19.0)	1,592 (19.7)
4	45,344,456 (28.7)	90,239 (21.3)	1,511 (32.0)	1,787 (22.2)
5	23,957,450 (15.2)	41,597 (9.8)	787 (16.7)	918 (11.4)
>5	15,109,618 (9.6)	20,814 (4.9)	438 (9.3)	674 (8.4)

Enrollee: Individual who is enrolled in an included health insurance plan.

The results of the stratified regression analyses for the model assessing exposure to family member(s) with MRSA are presented in Table 2. A total of 1,403,464 strata were created based on combinations of demographic, enrollment, and risk factor characteristics. A significant association was observed with prior exposure to family member(s) with MRSA. The estimated incidence rate of MRSA among those who were exposed to a family member with an MRSA diagnosis was over 70 times the rate compared to those who were not exposed to a family member with an MRSA diagnosis (IRR: 71.03 [95% CI, 67.73–74.50], *P* < .001). Prior hospitalization of an individual was also associated with an increased risk of MRSA (IRR: 3.54, [95% CI, 3.47–3.61], *P* < .001). Additionally, consistent with prior findings, increasing number of comorbidities, prior antimicrobial drug use, and presence of an infant <2 years of age in the household were associated with increased risk of MRSA. We also found that risk peaked in 2009 (see Supplementary Table 2.)

**Table 2.** Results of regression analysis for prior exposure to family member(s) with MRSA using a quasi-Poisson model^a^

Variable	IRR (95% Confidence interval)	*P* value
**Prior exposure to family member(s) with MRSA**	71.03 (67.73, 74.50)	<0.001
**Prior hospitalization**	3.54 (3.47, 3.61)	<0.001
**Age group (years)**		
0-17	REF	REF
18-40	1.22 (1.20, 1.24)	<0.001
41-65	1.19 (1.17, 1.21)	<0.001
>65	1.31 (1.28, 1.34)	<0.001
**Sex**		
Male	REF	REF
Female	0.80 (0.79, 0.80)	<0.001
**Total number of comorbidities within 60 days**		
0	REF	REF
1	1.82 (1.80, 1.85)	<0.001
2	3.23 (3.17, 3.29)	<0.001
3	5.51 (5.38, 5.64)	<0.001
4	8.40 (8.17, 8.64)	<0.001
5	11.83 (11.44, 12.23)	<0.001
6	15.38 (14.78, 16.01)	<0.001
7	19.81 (18.89, 20.78)	<0.001
8	24.65 (23.26, 26.13)	<0.001
9	30.52 (28.39, 32.82)	<0.001
10	36.32 (33.00, 39.98)	<0.001
11	40.89 (35.75, 46.76)	<0.001
12	42.99 (35.07, 52.69)	<0.001
13	47.96 (34.67, 66.34)	<0.001
14	47.70 (26.90, 84.59)	<0.001
≥15	50.02 (19.64, 127.40)	<0.001
**Outpatient antimicrobial prescription within 60 days**	8.14 (8.05, 8.22)	<0.001
**Infant age <2 y in family**	1.15 (1.13, 1.17)	<0.001

IRR, Incidence Rate Ratio.

Note. Models were also adjusted for year, month, and family size. Effect estimates for these covariates can be found in Supplementary Table 2.

a The regression model included an offset for number of enrollment months. Given that the prior family exposure group was followed for 30 days to identify secondary MRSA cases, the length of their enrollment period is 30 days. For the unexposed group, the length of enrollment was the length of a given month.

The results of the stratified regression analyses for the model assessing prior exposure to hospitalized family members are presented in Table 3. A total of 779,353 strata were created based on different combinations of demographic, enrollment, and risk factor characteristics. In the model utilizing a single indicator for exposure to a hospitalized family member, we found that the IRR of MRSA among enrollees exposed to a recently hospitalized family member(s) was 1.44 (95% CI, 1.39–1.49). Similarly, we observed a dose-response relationship between the incidence rate of MRSA and the amount of time family members spent hospitalized in the prior 30 days. Using 0 days as the reference category, the IRR of MRSA increased from 1.34 (95% CI, 1.28–1.40) for 1–3 days family members spent hospitalized to 1.49 (95% CI, 1.41–1.57) for 4–10 days family members spent hospitalized (Table 3); the IRR then remained elevated at around 1.7–1.8 for stays beyond 10 days. Consistent with prior findings, increasing number of comorbidities, prior antimicrobial drug use, and presence of an infant <2 years of age in the household were associated with increased risk of MRSA. Finally, we found that risk for MRSA was significantly higher in all years after 2001, but the peak occurred around 2010. (See Supplemental Table 3.)

**Table 3.** Results of regression analysis for prior exposure to hospitalized family member(s) using quasi-Poisson models^a^

Variable	Family member spent any time in hospital		Duration of time family member spent in hospital	
	IRR (95% Confidence Interval)	*P* value	IRR (95% Confidence Interval)	*P* value
**Hospitalized family member in prior 30-days**	1.44 (1.39, 1.49)	<0.001	–	–
**Days family member(s) spent hospitalized in prior 30-days**				
0	–	–	REF	REF
1–3	–	–	1.34 (1.28, 1.40)	<0.001
4–10	–	–	1.49 (1.41, 1.57)	<0.001
11–20	–	–	1.82 (1.63, 2.03)	<0.001
21–30	–	–	1.79 (1.49, 2.16)	<0.001
>30	–	–	1.74 (1.49, 2.04)	<0.001
**Age group (years)**				
0-17	REF	REF	REF	REF
18-40	1.23 (1.21, 1.25)	<0.001	1.23 (1.21, 1.24)	<0.001
41-65	1.16 (1.14, 1.17)	<0.001	1.16 (1.14, 1.17)	<0.001
>65	1.30 (1.28, 1.33)	<0.001	1.30 (1.28, 1.33)	<0.001
**Sex**				
Male	REF	REF	REF	REF
Female	0.80 (0.79, 0.81)	<0.001	0.80 (0.79, 0.81)	<0.001
**Total number of comorbidities within 60 days**				
0	REF	REF	REF	REF
1	1.77 (1.75, 1.79)	<0.001	1.77 (1.75, 1.79)	<0.001
2	3.22 (3.17, 3.28)	<0.001	3.22 (3.17, 3.27)	<0.001
3	6.00 (5.88, 6.12)	<0.001	6.00 (5.88, 6.11)	<0.001
4	10.74 (10.47, 11.01)	<0.001	10.73 (10.49, 10.99)	<0.001
5	18.07 (17.52, 18.64)	<0.001	18.07 (17.56, 18.59)	<0.001
6	27.81 (26.78, 28.89)	<0.001	27.81 (26.86, 28.79)	<0.001
7	42.13 (40.23, 44.11)	<0.001	42.11 (40.38, 43.92)	<0.001
8	58.82 (55.52, 62.32)	<0.001	58.80 (55.78, 61.99)	<0.001
9	78.75 (73.11, 84.83)	<0.001	78.73 (73.55, 84.26)	<0.001
10	100.27 (90.67, 110.90)	<0.001	100.24 (91.42, 109.90)	<0.001
11	132.09 (115.10, 151.59)	<0.001	132.02 (116.41, 149.73)	<0.001
12	149.85 (121.42, 184.93)	<0.001	149.73 (123.53, 181.47)	<0.001
13	151.78 (105.83, 217.67)	<0.001	151.67 (109.08, 210.89)	<0.001
14	133.12 (66.76, 265.44)	<0.001	133.16 (70.86, 250.24)	<0.001
≥15	142.30 (47.79, 423.65)	<0.001	142.54 (52.58, 386.45)	<0.001
**Outpatient antimicrobial prescription within 60 days**	9.03 (8.95, 9.11)	<0.001	9.03 (8.95, 9.10)	<0.001
**Infant age <2 y in family**	1.16 (1.14, 1.18)	<0.001	1.16 (1.14, 1.18)	<0.001

IRR, Incidence rate ratio.

Note. Models were adjusted for year, month, and family size. Effect estimates for these covariates can be found in Supplementary Table 3.

a The regression models included an offset for number of enrollment months. Given that the family hospitalization exposure group was followed for 30 days to identify secondary MRSA cases, the length of their enrollment period is 30 days. For the unexposed group, the length of enrollment was the length of a given month.

### Sensitivity analyses

Supplementary Tables 4 and 5 present the results of the sensitivity analyses where we expanded the exposure window to 60 days. For both study objectives, we found that using a 60-day exposure period had little impact on the effect estimates for the 1–30-day window, while the effect estimates during the 31–60-day window were attenuated. Specifically, the IRR for exposure to a family member with MRSA was 71.54 (95% CI, 68.20–75.04) and 33.61 (95% CI, 30.92–36.53) for the 1–30 versus 31–60 day window, respectively. Additionally, the IRR for exposure to a recently hospitalized family member was 1.45 (95% CI, 1.40–1.50) and 1.32 (95% CI, 1.27–1.37) for the 1–30 versus 31–60 day window, respectively.

## Discussion

In this study, we examined the risk of an MRSA infection among household members with exposure to either a family member with a recent MRSA infection or a family member recently discharged from a hospital without an MRSA diagnosis. Not surprisingly, we found that an individual’s risk of MRSA infections increased when one or more household members were directly exposed to a family member with MRSA. However, we also found that risk increased when one or more household members were discharged from a hospital without an MRSA diagnosis, suggesting that some proportion of patients without an MRSA diagnosis are likely colonized with MRSA during their hospital stay. Furthermore, we found the rate of MRSA infections in such households increased as the length of the stay for the recently hospitalized family member increased. Other factors associated with MRSA infections among household members included number of comorbidities, prior antibiotic usage, and the presence of young children in the family.

The three MRSA exposure effects we estimated (ie, hospital, MRSA-diagnosed family member, and recently discharged family member) are consistent with the broader understanding of MRSA transmission. In our primary model, exposure to family member diagnosed with MRSA had a large association with incidence (IRR: 71.03 [95% CI, 67.73–74.50]) while exposure to a hospital setting had a more moderate association (IRR: 3.54 [95% CI, 3.47–3.61]). The relative magnitude of these estimates is consistent with the fact that hospital settings carry some risk of exposure to MRSA, but with a lower probability than living with someone who has recently been infected.^29^ People colonized with MRSA but not diagnosed with infection can also transmit MRSA, and to isolate this effect we removed individuals who were themselves hospitalized or had a family member diagnosed with MRSA. We then found that exposure to a recently discharged family member without an MRSA diagnosis was also associated with an increase in MRSA incidence, but to slightly lesser degree than hospitalization (IRR: 1.44 [95% CI, 1.39–1.49]). This relatively smaller estimate is consistent with our understanding that only a portion of hospitalized patients are colonized with MRSA at discharge.^30^ Nevertheless, it is worth considering that this effect size, and the relatively limited number of such transmission events we can observe may not fully capture the population-level impact of patients colonized with MRSA on discharge. There are a large number of hospital discharges in the community, yet we only capture a portion of all postdischarge exposures. In particular, we are only able to observe a subset of the household members in our population who are enrolled in the same insurance plan, we cannot observe colonization events not resulting in infection, and we cannot observe other settings (eg, long-term care) where similar events may occur. There were likely many individuals colonized and infected that we could not observe in our analysis. Thus, this exposure pathway may still represent a significant reservoir for sustaining MRSA infections within the community.

The MRSA household transmission risk associated with hospital stays has multiple implications. First, we identified an important potential link between hospitals and community cases of MRSA. Second, our results highlight how interventions designed to control MRSA in hospitals may impact the spread of MRSA in the community. This link between hospitals in households may also have important implications for MRSA surveillance. Finally, family history has long been recognized as important component of the medical record. Our results suggest that a family member’s healthcare exposure may be important for clinicians to consider. Indeed, hospital discharges with or without a diagnosis of *C. difficile* are associated with an increased risk for *C. difficile* infections among household family members.^27,28^

Multiple studies describe households as a reservoir for MRSA and the propensity of cases to “ping-pong” between household members.^12,24,31^ Studies have also identified how environmental contamination from fabric, including towels and bedding from infected patients, may facilitate additional or recurrent cases within a household.^32–34^ However, most of these investigations have started with an index case—a family member with an active infection.^35^ In contrast, our approach allowed us to explore recently hospitalized patients as a potential source for household cases of MRSA. With our approach, we cannot directly measure colonization among discharged patients. However, assessing colonization using traditional approaches is also difficult because it can be intermittent and difficult to ascertain if multiple body sites are not assessed.^10,35,36^ For example, relying upon nasal colonization alone is known to lead to underestimates of MRSA colonization.^10,35^ Furthermore, traditional studies of colonization and household transmission are substantially more expensive than our approach.

This study is subject to multiple limitations which suggest targets for future investigation. First, we cannot establish the exact sizes of the families examined nor the exact exposure status among family members. Families who share the same health insurance policy might not live together (eg, students in college) and family members living together might be covered by separate health insurance policies. Thus, some of the exposures and non-exposures we identified may be misclassified. Second, we use administrative data and rely on diagnostic codes to determine MRSA diagnoses and comorbidities. These codes have varying sensitivity and specificity.^37,38^ We do not have access to sequencing data used in some previous work to study potential transmission events. Moreover, we cannot determine the exact exposure period or colonization using billing data alone. Third, we did not differentiate between different types of MRSA infections (eg, blood stream infections, soft-tissue infections, etc). Different types of infections have varying degrees of transmissibility. Fourth, because we used data from a commercially insured population, it is difficult to generalize to other populations. Fifth, we cannot determine the strains represented in our sample, but the risk for MRSA peaked around 2009–2010 and fell thereafter. Finally, household transmission of methicillin-sensitive Staphylococcus aureus may exhibit similar household effects but was not the focus of this work; this should be considered in future work.

In conclusion, despite the limitations with our approach, we showed a substantial association between hospitalized patients returning home and an increased risk of MRSA infections among their family members. Because approximately 25 million people have overnight hospital stays each year in the United States,^39^ patients recently discharged from hospitals represent a potentially major source of MRSA infections among household members.

## Supporting information

Miller et al. supplementary material Miller et al. supplementary material

## Supplementary material

To view supplementary material for this article, please visit https://doi.org/10.1017/ice.2024.106
