# Supplementary material for: Hospitalizations among family members increase the risk of MRSA infection in a household
Source: Infect Control Hosp Epidemiol. 2024 Aug 7;45(7):826–32. doi: 10.1017/ice.2024.106 (PMC11439592; doi:10.1017/ice.2024.106)

**Supplementary Material**

**Supplementary Table 1** – ICD-9 and ICD-10 codes used to identify cases of MRSA

| **ICD Group** | **Code** | **Description** |
| --- | --- | --- |
| ICD-9 | 038.12 | Sepsis due to Methicillin resistant Staphylococcus aureus |
|  | 041.12 | Methicillin resistant Staphylococcus aureus in conditions classified elsewhere and of unspecified site |
|  | 482.42 | Pneumonia due to Methicillin resistant Staphylococcus aureus |
|  | V09.0 | Infection with drug-resistant microorganisms, unspecified, without mention of multiple drug resistance |
| ICD-10 | A41.02 | Sepsis due to Methicillin resistant Staphylococcus aureus |
|  | J15.212 | Pneumonia due to Methicillin resistant Staphylococcus aureus |
|  | B95.62 | Methicillin resistant Staphylococcus aureus infection as the cause of diseases classified elsewhere |
|  | A49.02 | Methicillin resistant Staphylococcus aureus infection, unspecified site |

**Supplementary Table 2** - Results of Regression Analysis for Prior Exposure to Family Member(s) with MRSA using Quasi-Poisson Models for the remaining variables not reported in Table 2

| **Variable** | **IRR**  **(95% Confidence Interval)** | **P-value** |
| --- | --- | --- |
| **Year** |  |  |
| 2001 | REF | REF |
| 2002 | 1.94 (1.55, 2.42) | <0.001 |
| 2003 | 2.75 (2.22, 3.39) | <0.001 |
| 2004 | 3.92 (3.20, 4.82) | <0.001 |
| 2005 | 5.73 (4.68, 7.01) | <0.001 |
| 2006 | 8.25 (6.75, 10.08) | <0.001 |
| 2007 | 11.78 (9.65, 14.37) | <0.001 |
| 2008 | 14.87 (12.19, 18.14) | <0.001 |
| 2009 | 20.33 (16.67, 24.79) | <0.001 |
| 2010 | 20.06 (16.45, 24.46) | <0.001 |
| 2011 | 19.65 (16.11, 23.96) | <0.001 |
| 2012 | 18.68 (15.32, 22.78) | <0.001 |
| 2013 | 17.52 (14.36, 21.37) | <0.001 |
| 2014 | 17.34 (14.22, 21.15) | <0.001 |
| 2015 | 13.81 (11.31, 16.85) | <0.001 |
| 2016 | 12.79 (10.48, 15.60) | <0.001 |
| 2017 | 11.95 (9.79, 14.59) | <0.001 |
| 2018 | 11.06 (9.05, 13.50) | <0.001 |
| 2019 | 10.14 (8.30, 12.39) | <0.001 |
| 2020 | 9.54 (7.80, 11.66) | <0.001 |
| 2021 | 9.16 (7.49, 11.20) | <0.001 |
| **Month** |  |  |
| January | REF | REF |
| February | 0.88 (0.86, 0.90) | <0.001 |
| March | 0.84 (0.82, 0.86) | <0.001 |
| April | 0.85 (0.83, 0.87) | <0.001 |
| May | 0.85 (0.83, 0.87) | <0.001 |
| June | 0.98 (0.96, 1.01) | 0.12 |
| July | 1.03 (1.01, 1.06) | 0.01 |
| August | 1.14 (1.11, 1.16) | <0.001 |
| September | 1.12 (1.09, 1.15) | <0.001 |
| October | 1.07 (1.04, 1.09) | <0.001 |
| November | 0.94 (0.91, 0.96) | <0.001 |
| December | 0.84 (0.82, 0.86) | <0.001 |
| **Family Size** |  |  |
| 2 | REF | REF |
| 3 | 0.91 (0.90, 0.92) | <0.001 |
| 4 | 0.81 (0.80, 0.83) | <0.001 |
| 5 | 0.84 (0.82, 0.86) | <0.001 |
| >5 | 0.89 (0.87, 0.91) | <0.001 |

**Supplementary Table 3.** Results of Regression Analysis for Prior Exposure to Hospitalized Family Member(s) using Quasi-Poisson Models for the remaining variables not reported in Table 3

| **Variable** | **Family member spent any time in hospital** | | **Duration of time family member spent in hospital** | |
| --- | --- | --- | --- | --- |
|  | **IRR**  **(95% Confidence Interval)** | **P-value** | **IRR**  **(95% Confidence Interval)** | **P-value** |
| **Year** |  |  |  |  |
| 2001 | REF | REF | REF | REF |
| 2002 | 2.15 (1.72, 2.69) | <0.001 | 2.15 (1.75, 2.64) | <0.001 |
| 2003 | 3.14 (2.54, 3.88) | <0.001 | 3.14 (2.59, 3.81) | <0.001 |
| 2004 | 4.72 (3.84, 5.79) | <0.001 | 4.72 (3.91, 5.69) | <0.001 |
| 2005 | 7.20 (5.88, 8.82) | <0.001 | 7.20 (5.98, 8.67) | <0.001 |
| 2006 | 10.67 (8.72, 13.05) | <0.001 | 10.67 (8.87, 12.83) | <0.001 |
| 2007 | 15.44 (12.63, 18.88) | <0.001 | 15.45 (12.85, 18.56) | <0.001 |
| 2008 | 19.71 (16.13, 24.09) | <0.001 | 19.72 (16.42, 23.68) | <0.001 |
| 2009 | 26.96 (22.07, 32.94) | <0.001 | 26.97 (22.46, 32.38) | <0.001 |
| 2010 | 26.72 (21.87, 32.65) | <0.001 | 26.73 (22.26, 32.09) | <0.001 |
| 2011 | 26.09 (21.36, 31.88) | <0.001 | 26.10 (21.73, 31.33) | <0.001 |
| 2012 | 24.79 (20.29, 30.29) | <0.001 | 24.79 (20.65, 29.77) | <0.001 |
| 2013 | 22.99 (18.81, 28.08) | <0.001 | 22.99 (19.14, 27.61) | <0.001 |
| 2014 | 22.80 (18.66, 27.86) | <0.001 | 22.80 (18.99, 27.38) | <0.001 |
| 2015 | 17.81 (14.57, 21.77) | <0.001 | 17.81 (14.82, 21.39) | <0.001 |
| 2016 | 16.42 (13.43, 20.07) | <0.001 | 16.42 (13.67, 19.73) | <0.001 |
| 2017 | 15.41 (12.61, 18.84) | <0.001 | 15.41 (12.82, 18.52) | <0.001 |
| 2018 | 14.19 (11.60, 17.35) | <0.001 | 14.19 (11.81, 17.05) | <0.001 |
| 2019 | 12.95 (10.59, 15.84) | <0.001 | 12.95 (10.77, 15.57) | <0.001 |
| 2020 | 12.14 (9.92, 14.86) | <0.001 | 12.14 (10.10, 14.60) | <0.001 |
| 2021 | 11.71 (9.56, 14.33) | <0.001 | 11.70 (9.72, 14.07) | <0.001 |
| **Month** |  |  |  |  |
| January | REF | REF | REF | REF |
| February | 0.87 (0.85, 0.89) | <0.001 | 0.87 (0.85, 0.88) | <0.001 |
| March | 0.82 (0.81, 0.84) | <0.001 | 0.82 (0.81, 0.84) | <0.001 |
| April | 0.84 (0.82, 0.86) | <0.001 | 0.84 (0.82, 0.85) | <0.001 |
| May | 0.84 (0.83, 0.86) | <0.001 | 0.84 (0.83, 0.86) | <0.001 |
| June | 0.98 (0.96, 1.00) | 0.04 | 0.98 (0.96, 1.00) | 0.02 |
| July | 1.03 (1.01, 1.06) | <0.01 | 1.03 (1.01, 1.05) | <0.001 |
| August | 1.14 (1.12, 1.17) | <0.001 | 1.14 (1.12, 1.16) | <0.001 |
| September | 1.13 (1.11, 1.15) | <0.001 | 1.13 (1.11, 1.15) | <0.001 |
| October | 1.07 (1.05, 1.09) | <0.001 | 1.07 (1.05, 1.09) | <0.001 |
| November | 0.93 (0.91, 0.95) | <0.001 | 0.93 (0.91, 0.95) | <0.001 |
| December | 0.83 (0.81, 0.85) | <0.001 | 0.83 (0.81, 0.85) | <0.001 |
| **Family Size** |  |  |  |  |
| 2 | REF | REF | REF | REF |
| 3 | 0.91 (0.90, 0.93) | <0.001 | 0.91 (0.90, 0.93) | <0.001 |
| 4 | 0.82 (0.81, 0.83) | <0.001 | 0.82 (0.81, 0.83) | <0.001 |
| 5 | 0.85 (0.83, 0.86) | <0.001 | 0.85 (0.83, 0.86) | <0.001 |
| >5 | 0.90 (0.88, 0.92) | <0.001 | 0.90 (0.88, 0.92) | <0.001 |

**Supplementary Table 4.** Sensitivity Analysis - Results of Regression Analysis for Prior Exposure to Family Member(s) with MRSA using Quasi-Poisson Models

| **Variable** | **IRR**  **(95% Confidence Interval)** | **P-value** |
| --- | --- | --- |
| **Prior exposure to family member(s) with MRSA** |  |  |
| 1-30 days | 71.54 (68.20, 75.04) | <0.001 |
| 31-60 days | 33.61 (30.92, 36.53) | <0.001 |
| **Prior hospitalization** |  |  |
| 1-30 days | 4.10 (4.02, 4.19) | <0.001 |
| 31-60 days | 2.18 (2.12, 2.23) | <0.001 |
| **Age group (years)** |  |  |
| 0-17 | REF | REF |
| 18-40 | 1.22 (1.20, 1.24) | <0.001 |
| 41-65 | 1.19 (1.18, 1.21) | <0.001 |
| >65 | 1.31 (1.28, 1.33) | <0.001 |
| **Sex** |  |  |
| Male | REF | REF |
| Female | 0.80 (0.79, 0.80) | <0.001 |
| **Total number of** **comorbidities within 60 days** |  |  |
| 0 | REF | REF |
| 1 | 1.79 (1.76, 1.81) | <0.001 |
| 2 | 3.05 (2.99, 3.11) | <0.001 |
| 3 | 4.96 (4.84, 5.08) | <0.001 |
| 4 | 7.19 (6.99, 7.40) | <0.001 |
| 5 | 9.76 (9.43, 10.10) | <0.001 |
| 6 | 12.37 (11.88, 12.88) | <0.001 |
| 7 | 15.68 (14.95, 16.46) | <0.001 |
| 8 | 19.35 (18.24, 20.52) | <0.001 |
| 9 | 23.91 (22.23, 25.72) | <0.001 |
| 10 | 28.40 (25.78, 31.27) | <0.001 |
| 11 | 32.01 (27.97, 36.63) | <0.001 |
| 12 | 33.92 (27.65, 41.61) | <0.001 |
| 13 | 38.08 (27.49, 52.74) | <0.001 |
| 14 | 37.41 (21.05, 66.47) | <0.001 |
| $\geq$15 | 39.48 (15.45, 100.90) | <0.001 |
| **Outpatient antimicrobial prescription within 60 days** | 8.09 (8.00, 8.17) | <0.001 |
| **Infant age <2 y in family** | 1.12 (1.09, 1.14) | <0.001 |
| **Year** |  |  |
| 2001 | REF | REF |
| 2002 | 1.94 (1.55, 2.42) | <0.001 |
| 2003 | 2.75 (2.22, 3.40) | <0.001 |
| 2004 | 3.93 (3.20, 4.82) | <0.001 |
| 2005 | 5.73 (4.68, 7.02) | <0.001 |
| 2006 | 8.27 (6.77, 10.12) | <0.001 |
| 2007 | 11.80 (9.66, 14.41) | <0.001 |
| 2008 | 14.91 (12.21, 18.20) | <0.001 |
| 2009 | 20.48 (16.78, 24.99) | <0.001 |
| 2010 | 20.23 (16.57, 24.68) | <0.001 |
| 2011 | 19.84 (16.26, 24.22) | <0.001 |
| 2012 | 18.90 (15.49, 23.07) | <0.001 |
| 2013 | 17.76 (14.55, 21.68) | <0.001 |
| 2014 | 17.63 (14.44, 21.51) | <0.001 |
| 2015 | 14.08 (11.53, 17.19) | <0.001 |
| 2016 | 13.07 (10.70, 15.96) | <0.001 |
| 2017 | 12.22 (10.01, 14.93) | <0.001 |
| 2018 | 11.31 (9.26, 13.82) | <0.001 |
| 2019 | 10.39 (8.50, 12.71) | <0.001 |
| 2020 | 9.78 (8.00, 11.96) | <0.001 |
| 2021 | 9.41 (7.69, 11.51) | <0.001 |
| **Month** |  |  |
| January | REF | REF |
| February | 0.88 (0.86, 0.90) | <0.001 |
| March | 0.84 (0.82, 0.86) | <0.001 |
| April | 0.85 (0.83, 0.87) | <0.001 |
| May | 0.85 (0.83, 0.87) | <0.001 |
| June | 0.98 (0.96, 1.01) | 0.13 |
| July | 1.03 (1.01, 1.06) | 0.01 |
| August | 1.13 (1.11, 1.16) | <0.001 |
| September | 1.12 (1.09, 1.15) | <0.001 |
| October | 1.06 (1.04, 1.09) | <0.001 |
| November | 0.94 (0.92, 0.96) | <0.001 |
| December | 0.84 (0.82, 0.86) | <0.001 |
| **Family Size** |  |  |
| 2 | REF | REF |
| 3 | 0.91 (0.90, 0.92) | <0.001 |
| 4 | 0.81 (0.80, 0.82) | <0.001 |
| 5 | 0.84 (0.82, 0.85) | <0.001 |
| >5 | 0.88 (0.86, 0.91) | <0.001 |

**Supplementary Table 5.** Sensitivity Analysis - Results of Regression Analysis for Prior Exposure to Hospitalized Family Member(s) using Quasi-Poisson Models

| **Variable** | **Family member spent any time in hospital** | |
| --- | --- | --- |
|  | **IRR**  **(95% Confidence Interval)** | **P-value** |
| **Hospitalized family member in prior** |  |  |
| 1-30 days | 1.45 (1.40, 1.50) | <0.001 |
| 31-60 days | 1.32 (1.27, 1.37) | <0.001 |
| **Age group (years)** |  |  |
| 0-17 | REF | REF |
| 18-40 | 1.23 (1.21, 1.24) | <0.001 |
| 41-65 | 1.16 (1.14, 1.17) | <0.001 |
| >65 | 1.30 (1.27, 1.32) | <0.001 |
| **Sex** |  |  |
| Male | REF | REF |
| Female | 0.80 (0.79, 0.81) | <0.001 |
| **Total number of** **comorbidities within 60 days** |  |  |
| 0 | REF | REF |
| 1 | 1.77 (1.75, 1.79) | <0.001 |
| 2 | 3.22 (3.17, 3.27) | <0.001 |
| 3 | 5.99 (5.87, 6.11) | <0.001 |
| 4 | 10.72 (10.46, 10.98) | <0.001 |
| 5 | 18.04 (17.51, 18.58) | <0.001 |
| 6 | 27.76 (26.77, 28.78) | <0.001 |
| 7 | 42.03 (40.24, 43.91) | <0.001 |
| 8 | 58.69 (55.57, 62.00) | <0.001 |
| 9 | 78.56 (73.21, 84.30) | <0.001 |
| 10 | 100.00 (90.89, 110.03) | <0.001 |
| 11 | 131.72 (115.59, 150.10) | <0.001 |
| 12 | 149.48 (122.43, 182.50) | <0.001 |
| 13 | 151.33 (107.48, 213.06) | <0.001 |
| 14 | 132.45 (68.81, 254.93) | <0.001 |
| $\geq$15 | 142.14 (50.48, 400.21) | <0.001 |
| **Outpatient antimicrobial prescription within 60 days** | 9.03 (8.95, 9.11) | <0.001 |
| **Infant age <2 y in family** | 1.15 (1.13, 1.17) | <0.001 |
| **Year** |  |  |
| 2001 | REF | REF |
| 2002 | 2.15 (1.74, 2.66) | <0.001 |
| 2003 | 3.14 (2.57, 3.84) | <0.001 |
| 2004 | 4.72 (3.88, 5.73) | <0.001 |
| 2005 | 7.20 (5.94, 8.73) | <0.001 |
| 2006 | 10.67 (8.81, 12.92) | <0.001 |
| 2007 | 15.45 (12.77, 18.69) | <0.001 |
| 2008 | 19.72 (16.31, 23.85) | <0.001 |
| 2009 | 26.97 (22.31, 32.62) | <0.001 |
| 2010 | 26.74 (22.11, 32.33) | <0.001 |
| 2011 | 26.10 (21.59, 31.56) | <0.001 |
| 2012 | 24.80 (20.51, 29.99) | <0.001 |
| 2013 | 23.00 (19.02, 27.81) | <0.001 |
| 2014 | 22.82 (18.87, 27.59) | <0.001 |
| 2015 | 17.82 (14.73, 21.56) | <0.001 |
| 2016 | 16.43 (13.58, 19.88) | <0.001 |
| 2017 | 15.42 (12.74, 18.66) | <0.001 |
| 2018 | 14.20 (11.73, 17.19) | <0.001 |
| 2019 | 12.96 (10.70, 15.69) | <0.001 |
| 2020 | 12.16 (10.04, 14.72) | <0.001 |
| 2021 | 11.72 (9.67, 14.19) | <0.001 |
| **Month** |  |  |
| January | REF | REF |
| February | 0.87 (0.85, 0.88) | <0.001 |
| March | 0.82 (0.81, 0.84) | <0.001 |
| April | 0.84 (0.82, 0.85) | <0.001 |
| May | 0.84 (0.83, 0.86) | <0.001 |
| June | 0.98 (0.96, 1.00) | 0.03 |
| July | 1.03 (1.01, 1.05) | <0.001 |
| August | 1.14 (1.12, 1.16) | <0.001 |
| September | 1.13 (1.11, 1.15) | <0.001 |
| October | 1.07 (1.05, 1.09) | <0.001 |
| November | 0.93 (0.91, 0.95) | <0.001 |
| December | 0.83 (0.81, 0.85) | <0.001 |
| **Family Size** |  |  |
| 2 | REF | REF |
| 3 | 0.91 (0.90, 0.93) | <0.001 |
| 4 | 0.82 (0.81, 0.83) | <0.001 |
| 5 | 0.84 (0.83, 0.86) | <0.001 |
| >5 | 0.90 (0.88, 0.92) | <0.001 |

**Supplementary Figure 1** – Trends in MRSA cases across time, broken down by prior family exposure


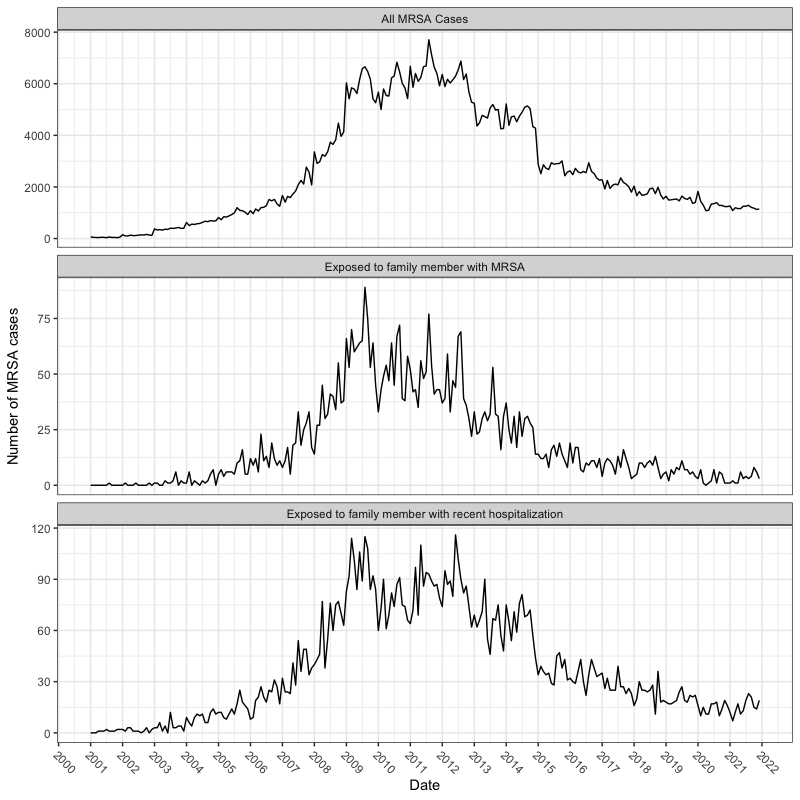

Supplement: Miller et al. supplementary material [file S0899823X24001065sup001.docx]
